# Co-infusion of haplo-identical CD19-chimeric antigen receptor T cells and stem cells achieved full donor engraftment in refractory acute lymphoblastic leukemia

**DOI:** 10.1186/s13045-016-0357-z

**Published:** 2016-11-25

**Authors:** Bo Cai, Mei Guo, Yao Wang, Yajing Zhang, Jun Yang, Yelei Guo, Hanren Dai, Changlin Yu, Qiyun Sun, Jianhui Qiao, Kaixun Hu, Hongli Zuo, Zheng Dong, Zechuan Zhang, Mingxing Feng, Bingxia Li, Yujing Sun, Tieqiang Liu, Zhiqing Liu, Yi Wang, Yajing Huang, Bo Yao, Weidong Han, Huisheng Ai

**Affiliations:** 1 Department of Hematology and Transplantation, Affiliated Hospital of Academy of Military Medical Sciences, 8 Dongdajie, Beijing, 100071 China; 2 Department of Immunology/Department of Bio-therapeutic, Institute of Basic Medicine, School of Life Sciences, Chinese PLA General Hospital, 28 Fuxing Road, Beijing, 100853 China

**Keywords:** Allogeneic anti-CD19 chimeric antigen receptor (CAR) T cells, Haplo-identical mobilized peripheral blood stem cell infusion, B cell acute lymphoblastic leukemia (B-ALL), Relapsed and refractory, Case report

## Abstract

**Background:**

Elderly patients with relapsed and refractory acute lymphoblastic leukemia (ALL) have poor prognosis. Autologous CD19 chimeric antigen receptor-modified T (CAR-T) cells have potentials to cure patients with B cell ALL; however, safety and efficacy of allogeneic CD19 CAR-T cells are still undetermined.

**Case presentation:**

We treated a 71-year-old female with relapsed and refractory ALL who received co-infusion of haplo-identical donor-derived CD19-directed CAR-T cells and mobilized peripheral blood stem cells (PBSC) following induction chemotherapy. Undetectable minimal residual disease by flow cytometry was achieved, and full donor cell engraftment was established. The transient release of cytokines and mild fever were detected. Significantly elevated serum lactate dehydrogenase, alanine transaminase, bilirubin and glutamic-oxalacetic transaminase were observed from days 14 to 18, all of which were reversible after immunosuppressive therapy.

**Conclusions:**

Our preliminary results suggest that co-infusion of haplo-identical donor-derived CAR-T cells and mobilized PBSCs may induce full donor engraftment in relapsed and refractory ALL including elderly patients, but complications related to donor cell infusions should still be cautioned.

**Trial registration:**

Allogeneic CART-19 for Elderly Relapsed/Refractory CD19+ ALL. NCT02799550

**Electronic supplementary material:**

The online version of this article (doi:10.1186/s13045-016-0357-z) contains supplementary material, which is available to authorized users.

## Background

The outcome of relapsed and/or refractory acute lymphoblastic leukemia (ALL) in elderly patients still remains poor. CD19-directed chimeric antigen receptor-modified T (CAR-T) cells exhibit powerful capability to eliminate leukemia cells and showed a high CR rate and curable effect in patients with relapsed ALL [1, 2]. However, patients who have extremely high leukemia burden may meet with difficulty in collecting sufficient T cells, and prolonged B cell aplasia is another problem. Although allogeneic CAR-T cells have potentials to overcome some limitations of autologous CAR-T cells, the efficiency and graft-*versus*-host disease (GVHD) in the non-transplant background are still undetermined [3].

We previously reported improved survival of leukemia patients who were treated by infusion of haplo-identical granulocyte colony-stimulating factor (G-CSF)-mobilized peripheral blood stem cells (G-PBSCs) following chemotherapy (microtransplantation), and limited GVHD was present [4, 5]. Emerging evidence has shown that donor-derived CAR-T cells functioned well after allogeneic stem cell transplantation (allo-SCT), which implied that donor G-PBSCs may support donor-derived CAR-T cell survival in the recipient [6]. However, the safety and efficacy of allogeneic CAR-T cells in combination with G-PBSCs for leukemia patients are unclear. To evaluate the feasibility of this strategy, we initiated a phase I clinical trial, and here, we report one elderly patient with relapsed and refractory B cell ALL using the co-infusion of haplo-identical donor-derived CD19-directed CAR-T cells and G-PBSCs and achieved full donor cell engraftment.

## Case presentation

**Fig. 1:**
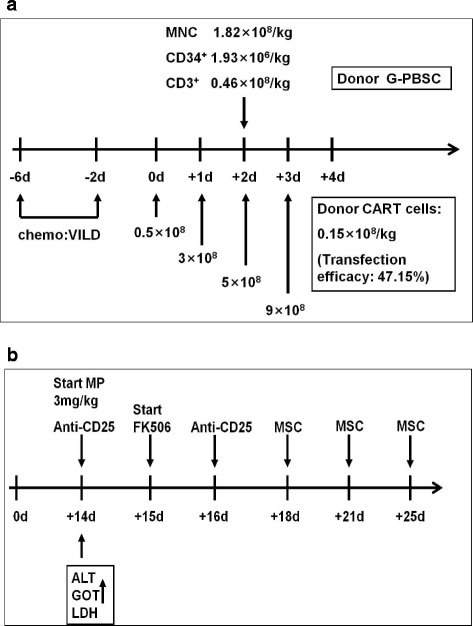
Protocol of haplo-identical CAR-T cell and G-PBSC infusions following chemotherapy and treatment for “GVHD-like” reaction. **a** Protocol of CAR-T and G-PBSC infusion in combination with chemotherapy. Chemotherapy included vindesine, idarubicin, pegaspargase, and dexamethasone. CAR-T cells were infused at a total dose of 0.32 × 10^8^/kg CD3^+^ cells (0.15 × 10^8^/kg CAR-T cells), and the numbers of mononuclear, CD34^+^ and CD3^+^ cells infused in the G-PBSCs were 1.82 × 10^8^/kg, 1.93 × 10^6^/kg, and 0.46 × 10^8^/kg, respectively. **b** Flow chart of treatment after developing “GVHD-like” reaction. Methylprednisolone at a dose of 3 mg/kg per day was administered intravenously right after the finding of liver dysfunction from day 14, and tacrolimus at a dose of 0.03 mg/kg per day was started intravenously on day 15. Anti-CD25 antibody at a dose of 25 mg per day was given on days 14 and 16. Mesenchymal stem cells with a number of 5 × 10^5^/kg per day were injected to the bone marrow cavity on days 18, 21, and 25

A 71-year-old female was admitted to the hospital because of pancytopenia. Complete blood count with manual differentiation showed 68% blasts, and bone marrow smear showed 68.5% blasts. Flow cytometry analysis revealed 41.7% cCD79a+, 97.2% CD19+, 69.5% CD20+, 54.5% CD10+, 97.3% CD38+, and 91.4% HLA-DR. She had normal female karyotype, and no fusion genes were detected. The primary diagnosis was ALL. She was treated with vindesine, mitoxantrone, cyclophosphamide, and dexamethasone as induction and achieved complete remission. A consolidation was given with the same regimen as induction. The patient suffered from fever and bone pain 2 months after the last chemotherapy, and repeated bone marrow smear showed 73% lymphoblasts. Two cycles of re-induction chemotherapy were initiated consisting of vindesine, fludarabine, cyclophosphamide, and prednisone; however, she failed to achieve the second remission. The bone marrow examination was repeated and showed 98% lymphoblasts with the same immunophenotype as primary diagnosis. Her karyotype shifted to 42-46, X, -X[11], add(2)(P25)[7], ?del(6)(q22)[8], -7[7], ?add(8)(q24)[3], +mar1-2[11][cp15] / 46, XX[7]. She was diagnosed with relapsed and refractory B cell ALL and was the first patient enrolled in this phase I clinical trial (NCT02799550). A re-induction chemotherapy was administered (vindesine, idarubicin, pegaspargase, and dexamethasone) which was followed by co-infusion of haplo-identical donor-derived CD19-directed CAR-T cells and G-PBSCs. Both CAR-T cells and G-PBSCs were from her son, who had 6/10 human leukocyte antigen (HLA) loci matched with the patient. Peripheral blood for CAR-T cell preparation was collected ahead of mobilization, and the materials and methods to produce, detect, and quantify CD19-directed CAR-T cells were reported previously [7] and detailed in Additional file 1. Mobilization and apheresis of donor peripheral mononuclear cells were described previously [5]. CAR-T cells were infused from the second to fifth day with escalating doses, and G-PBSCs were infused at the fourth day post chemotherapy (Fig. 1a).

### Response to treatment

**Fig. 2:**
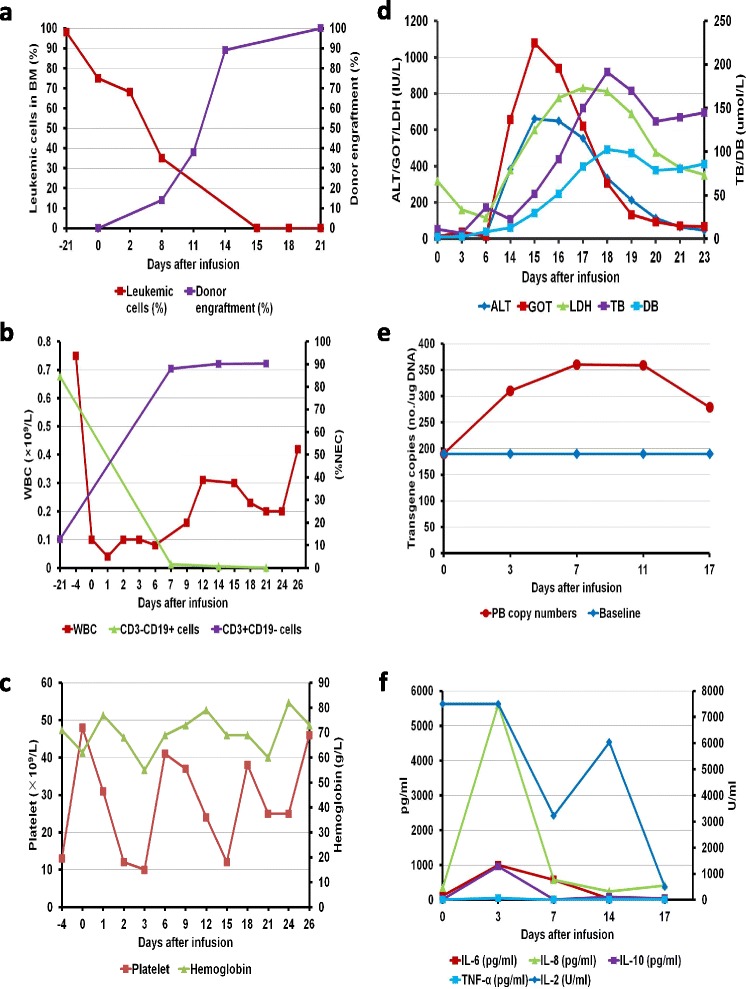
Clinical responses to infusions of haplo-identical CAR-T cells and G-PBSCs in the patient with ALL. **a** Leukemic cells in the bone marrow decreased and were finally undetected 15 days after the first CAR-T cell infusion. Meanwhile, the percentage of donor cells elevated to 100% 21 days after the first CAR-T cell infusion. **b** White blood cell count recovered slowly after the first CAR-T cell infusion, and the proportion of CD3^+^CD19^−^cells in nucleated cells in peripheral blood elevated to 90% and CD3^−^CD19^+^ cells decreased dramatically to 0.1% 21 days after the first CAR-T cell infusion. **c** Changes in the platelet count and hemoglobin level. **d** Elevated serum LDH, ALT, and GOT were detected on day 14. Levels of ALT and GOT achieved the peak on day 15. The level of LDH reached the peak on day 17. Elevations of TB and DB were also present following rises of LDH, ALT, and GOT and reached the peak on day 18. **e** Levels of the allogeneic CAR gene were monitored. The highest level in the peripheral blood was reached on day 7 with a copy number within twofold of the baseline. **f** Serum levels of cytokines were measured at the indicated time points before or after CAR-T cell and G-PBSC infusions. Levels of IL-6, IL-8, IL-10, and TNF-α elevated markedly on day 3 and then dropped quickly

Leukemic cells in the bone marrow were undetectable 15 days after the first CAR-T cell infusion (Fig. 2a), and minimal residual disease was also negative by flow cytometry in the bone marrow. Although the white blood cell count remained less than 0.5 × 10^9^/L 26 days after the first CAR-T cell infusion, the proportion of CD3^+^CD19^−^ cells in nucleated cells in peripheral blood elevated to 90% and CD3^−^CD19^+^ cells decreased dramatically to 0.1% 21 days after the first CAR-T cell infusion (Fig. 2b, c).

### Toxicity

The patient developed a fever (38.3 °C) with chill 1.5 h after the infusion of CAR-T cells on day 0, and the temperature returned to normal within 3 h without the administration of steroids and antibiotics. Intermittent fever occurred accompanied by a sharp elevation of C-reaction protein (peak value 336 mg/L on day 3) during the following days after the first infusion of CAR-T cells, and sepsis of *Escherichia coli* was confirmed through blood culture. Antibiotics were administrated and the fever was well controlled. No immediate infusion-related toxicity was noted during the infusion of CAR-T cells on days 1, 2, or 3.

Significantly elevated serum lactate dehydrogenase (LDH), alanine transaminase (ALT), glutamic-oxalacetic transaminase (GOT), and bilirubin were present from day 14, and the peak was observed and associated with skin rash in the neck area from days 15 to 18 (Fig. 2d). It was suspected as mild GVHD (no biopsy confirmation) although no severe diarrhea occurred. Methylprednisolone and tacrolimus were administered from days 14 and 15, respectively, and anti-CD25 antibody was given on days 14 and 16. As our previous study in haplo-identical non-myeloablative stem cell transplantation showed that intrabone marrow injection of mesenchymal stem cells (MSCs) could prevent severe acute GVHD [8], MSCs from an unrelated donor with a number of 5 × 10^5^/kg per day were injected directly to the bone marrow cavity on days 18, 21, and 25 (Fig. 1b). The materials and methods to produce MSCs were detailed in Additional file 2. The ALT, GOT, LDH, and bilirubin significantly decreased on days 18 and 19, respectively; however, bilirubin had a trend to elevate again from day 21 (Fig. 2d).

### Engraftment

We monitored donor cell engraftment in the peripheral blood and bone marrow by standard cytogenetic analysis and semi-quantitative polymerase chain reaction (PCR)-based analysis. Donor cells accounted for 14% of whole cells on day 8 after the first CAR-T cell infusion, which increased gradually to 100% on day 21 (Fig. 2a).

### Expansion of donor-derived CAR-T cells in vivo

The highest level of the allogeneic CAR gene in the peripheral blood was reached on day 7 after the first infusion of CAR-T cells with a copy number of 360 per ug of DNA which was within twofold of the baseline and associated with decrease of the percentage of CD3^−^CD19^+^ cells (Fig. 2b, e). The level of CAR dropped to 279 copies on day 17.

### Cytokine changes

Serum levels of IL-6, IL-8, IL-10, and TNF-α elevated markedly on day 3 after 3-day infusions of CAR-T cells (Fig. 2f). No signs of severe cytokine-release syndrome (CRS) or tumor lysis syndrome (TLS) were observed.

### Survival

The patient exhibited slight recovery of neutrophil count on day 26 after the first infusion of CAR-T cells; however, she refused any further treatment and was discharged on day 26 because of financial problems. She died from severe infection on day 31.

## Discussion and Conclusion

In this study, a 71-year-old relapsed and refractory ALL patient received co-infusion of haplo-identical donor-derived CD19-directed CAR-T cells and G-PBSCs and achieved full donor cell engraftment with mild toxicity.

It is speculated that allogeneic CAR-T cells may have potential benefit of reserving alloreactive-attacking capability to leukemic cells and help overcome limitation of autologous CAR-modified T cells [9]. However, it is unclear whether allogeneic CAR-T cells may persist and proliferate in vivo. In this patient, we persistently detected donor CAR-T cells within twofold of the baseline in host peripheral blood. These results suggested that allogeneic CAR-T cells can survive in vivo although their proliferative efficiency is much lower than that previously reported in which autologous CAR-T cell number amplifies thousands of times in vivo [1]. The anti-CD25 monoclonal antibody has been reported to eliminate donor-specific alloreactive T cells via blockade of the IL-2 binding site and then to prevent and treat acute GVHD [10, 11, 12]. However, no studies have investigated its influence to CAR-T cells. We treated this patient with anti-CD25 antibody on days 14 and 16, which was in accordance with the drop of the allogeneic CAR gene (Figs. 1b and 2e), implying the potential adverse impact of anti-CD25 antibody on CAR-T cell persistence and activation. It is also uncertain whether sustained pancytopenia in this patient suppressed proliferation of CD19-positive cells and consequently attenuated amplification activity of CAR-T cells. In fact, the persistence of CAR-T cells leads to prolonged B cell aplasia [1]. Allogeneic CAR-T cells may also cause donor/recipient-derived B cell clearance (Fig. 2b). Therefore, transient or low level expression of allogeneic CAR-T cells may facilitate donor/recipient-derived B cell reconstitution and simultaneously reserve anti-leukemic effect.

Another major concern is the safety of allogeneic CAR-T cells, particularly for severe GVHD. In this study, the patient developed obvious liver dysfunction and skin rash associated with donor cell engraftment, but no severe hypotension and polypnea were observed although mild elevated levels of cytokines including IL-6, IL-8, IL-10, and TNF-α were found. Therefore, we prefer to name it as a mild “GVHD-like” reaction instead of CRS in this patient. This reaction may correlate with low-intensity chemotherapy conditioning and CAR-T cell-mediated specific anti-leukemic action prior to the G-PBSC infusion, which suppressed patient’s normal immune function and consequently induced development of GVHD under the absence of GVHD prevention. This patient established full donor chimerism following a simple combined chemotherapy and infusions of allogeneic G-PBSCs and CD19-directed CAR-T cells, which may in part attribute to low proportion of recipient CD3^+^CD19^−^ T cells prior to cell infusions (Fig. 2b). Although a preclinical study reported CAR-T cells as a component of conditioning regimen of standard transplantation [13], whether allogeneic CAR-T cells improve engraftment is still undetermined. Further investigations will focus on a clinical study to evaluate the feasibility of this strategy in future.

## Additional files

Additional file 1: Preparation, detection, and quantification of CD19-directed chimeric antigen receptor-modified T (CAR-T) cells. (DOCX 17 kb)
Additional file 2: Preparation and quality control of mesenchymal stem cells (MSC). (DOCX 12 kb)

## Abbreviations

ALL: Acute lymphoblastic leukemia
allo-SCT: Allogeneic stem cell transplantation
ALT: Alanine transaminase
CAR-T cells: Chimeric antigen receptor-modified T cells
CRS: Cytokine-release syndrome
G-CSF: Granulocyte colony-stimulating factor
GOT: Glutamic-oxalacetic transaminase
G-PBSCs: G-CSF-mobilized peripheral blood stem cells
GVHD: Graft-*versus*-host disease
HLA: Human leukocyte antigen
LDH: Lactate dehydrogenase
MSC: Mesenchymal stem cells
PCR: Polymerase chain reaction
TLS: Tumor lysis syndrome
