# Supplementary material for: Co-infusion of haplo-identical CD19-chimeric antigen receptor T cells and stem cells achieved full donor engraftment in refractory acute lymphoblastic leukemia
Source: J Hematol Oncol. 2016 Nov 25;9:131. doi: 10.1186/s13045-016-0357-z (PMC5124292; doi:10.1186/s13045-016-0357-z)
Supplement: Additional file 1: — Preparation, detection, and quantification of CD19-directed chimeric antigen receptor-modified T (CAR-T) cells. (DOCX 17 kb) [file 13045_2016_357_MOESM1_ESM.docx]

**Constructs and lentivirus package**

The single chain fragment variable (scFv) sequence specific for CD19 was derived from HM852952.1 (GeneBank No.). CAR.19-4-1BBζ vectors harboring anti-CD19 scFv and human 4-1BB and CD3ζ signaling domains were generated. The cassettes were cloned into a lentiviral backbone. A pseudotyped, clinical-grade lentiviral vector was produced according to current good manufacturing practices. The green fluorescence protein (GFP) harboring vector CARCD137ζ-GFP was also constructed for verification of transduction efficiency.

**Generation and expansion of CAR T cells**

After the patient had given informed consent, 50 ml of blood was collected from each in evacuated tubes that contained heparin. Human PBMCs were isolated from fresh blood by Ficoll-Hypaque density gradient centrifugation. The PBMCs were washed three times, adjusted to a final concentration of 2×10^6^ cells/ml with medium (GT551,Takara, Japan) supplemented with 0.6% autogeneic serum, and then cultured in 75-cm^2^ culture flasks that had been coated with 8 ml of PBS that contained 5 μg/ml anti-human CD3 monoclonal antibody (Takara, Japan) at 4°C overnight. On day 0 of culture, added 500 U/ml recombinant human IL-2 (rhIL-2; PeproTech, USA) to the culture medium. The cells were cultured in a humidified 5% CO_2_ incubator at 37°C. Lentivirus-mediated CAR transduction was performed twice on day 2 and 3 of cell culture, respectively. The cells were transferred from the coated flasks to fresh flasks on day 4. Every three days, fresh medium and 500 U/ml rhIL-2 were added. Composition and purity were assessed by fluorescent-activated cell sorting (FACS) and were harvested beginning on days 10-12. Then, cells were harvested per flask, with a survival rate of >95%.

**Immunophenotyping**

Anti-human monoclonal antibodies against CD3, CD4, CD8, CD56, CD19, CD45RO, CD62L, and CCR7, were used for immunophenotyping analysis. All these antibodies and isotype-matched monoclonal antibodies were purchased from BD Biosciences (CA, USA). Data acquisition was performed using a FACSCalibur flow cytometer (BD Biosciences).

**Quantitative PCR**

We used real-time PCR to quantify the level of CAR transgenes as described previously.[^57^](#_ENREF_57) A 153-bp (base pair) fragment containing portions of the CD8a chain and adjacent 4-1BB chain was amplified. A standard curve was prepared for absolute quantitation of CAR transgene copies by making serial dilutions of the plasmid that encoded the CAR. An 7-point standard curve was generated consisting of 100 to 10^8^ copies/μl CAR plasmid spiked into 100 ng non-transduced control genomic DNA. Amplification of β-actin was used for normalization of DNA quantities.
