# Supplementary material for: Co-infusion of haplo-identical CD19-chimeric antigen receptor T cells and stem cells achieved full donor engraftment in refractory acute lymphoblastic leukemia
Source: J Hematol Oncol. 2016 Nov 25;9:131. doi: 10.1186/s13045-016-0357-z (PMC5124292; doi:10.1186/s13045-016-0357-z)
Supplement: Additional file 2: — Preparation and quality control of mesenchymal stem cells (MSC). (DOCX 12 kb) [file 13045_2016_357_MOESM2_ESM.docx]

Mesenchymal stem cells (MSCs) were isolated and propagated from bone marrow (BM) by puncturing the posterior iliac crest of the donor. All procedures were carried out under strict Good Manufacture Practice (GMP) criteria. BM aspirates were collected, mononuclear cells (MNC) were fractionated with a 1.077 g/mL Ficoll-Paque density gradient and then cultured in Dulbecco's Modified Eagle Medium (DMEM, Gibco, Rockville, MD) supplemented with 10% fetal bovine serum (FBS, Gibco) at 37℃ in a humidified environment containing 5% CO_2_. After culturing for 24 to 48 hours, nonadherent cells were removed and the adherent layer was further cultured until 70% to 80% confluence. Then MSCs were harvested and used at passages 3 to 5. The surface markers of MSCs were examined by a 4-color flow cytometer (EPICSxL-MCL, Beckman Coulter, Fullerton, CA), including Flk-1 (Santa Cruz Biotechnology, Santa Cruz, CA), CD29, CD34, CD44, CD45, CD105, and HLA-DR. Adipogenic differentiation was assessed by oil red O staining and osteogenic differentiation was analyzed by von Kossa method. MSCs were also examined for bacteria and mycoplasma contamination before use. They were resuspended in 0.5 mL of 0.9% sodium chloride and administrated by means of intrabone marrow injection into the posterior iliac crest of the patient.
